# Immunogenicity and safety of an adjuvanted inactivated polio vaccine, IPV-Al, following vaccination in children at **2, 4, 6** and at **15**–**18** months

**DOI:** 10.1016/j.vaccine.2020.02.066

**Published:** 2020-05-06

**Authors:** Xavier Sáez-Llorens, Birgit Thierry-Carstensen, Lina Saem Stoey, Charlotte Sørensen, Henrik Wachmann, Ananda S. Bandyopadhyay, Pernille Ingemann Nielsen, Mie Vestergaard Kusk

**Affiliations:** a Hospital del Niño Dr. José Renán Esquivel, Calle 34 Este, Panama City, Panama; b Cevaxin, Avenida Mexico Calle 33, Local 4, Calidonia, Panama; c National System of Investigation at Senacyt, Panama; d Statens Serum Institut, 5 Artillerivej, 2300 Copenhagen S, Denmark; e AJ Vaccines, 5 Artillerivej, 2300 Copenhagen S, Denmark; f Larix A/S, Lyskær 8b, 2730 Herlev, Denmark; g Bill & Melinda Gates Foundation, Seattle, WA, USA

**Keywords:** Affordable IPV, Standalone adjuvanted IPV, Aluminium hydroxide adjuvant, Booster vaccination, Immunogenicity, Polio, Primary vaccination

## Abstract

**Background:**

Availability of affordable inactivated polio vaccines (IPV) is of major importance to meet the increasing global supply needs. The results presented here demonstrate non-inferiority of a reduced-dose, aluminium hydroxide-adjuvanted IPV (IPV-Al) to standard IPV.

**Methods:**

A phase 3, observer-blinded, randomised, clinical trial was conducted in Panama in infants who received either IPV-Al (n = 400) or standard IPV (n = 400) at age 2, 4 and 6 months. In the booster trial, subjects received a single dose of IPV-Al at age 15–18 months. The primary endpoint was type-specific seroconversion, defined as an antibody titre ≥4-fold higher than the estimated maternal antibody titre and a titre ≥8, one month after the primary vaccination series. In the booster trial, the primary endpoint was the type-specific booster effects (geometric mean titre (GMT) post-booster (Day 28)/GMT pre-booster (Day 0).

**Results:**

Seroconversion rates following primary vaccination with IPV-Al vs IPV were: 96.1% vs 100% (type 1); 100% vs 100% (type 2); and 99.2% vs 100% (type 3) respectively. IPV-Al was non-inferior to IPV, as the lower 95% confidence limits of the treatment differences were above the pre-defined −10%-point limit: 3.94% (-6.51; −2.01) for type 1; 0.0% (-1.30; −1.37) for type 2; −0.85 (-2.46; 0.40) for type 3. The booster effects for the group primed with IPV-Al versus the group primed with IPV were 25.3 vs 9.2 (type 1), 19.1 vs 6.5 (type 2) and 50.4 vs 12.5 (type 3). IPV-Al had a comparable safety profile to that of IPV.

**Conclusions:**

Non-inferiority of IPV-Al to standard IPV with respect to seroconversion after vaccination at 2, 4 and 6 months was confirmed for all three poliovirus serotypes. A robust booster response was demonstrated following vaccination with IPV-Al, regardless of the primary vaccine received. Both vaccines were well tolerated.

ClinicalTrials.gov identifiers: NCT03025750 and NCT03671616.

Funding: Bill & Melinda Gates Foundation.

## 1 Introduction

More than 2.5 billion children have been vaccinated against polio since the start of the Global Polio Eradication Initiative (GPEI) in 1988. Since then, the global incidence of polio has dropped by >99% [1], with wild poliovirus (WPV) type 1 as the only source of all currently reported WPV infections [1], [2]. However, the number of WPV type 1 cases reported in Afghanistan and Pakistan has risen from 22 cases in 2017 to 33 in 2018. Furthermore, 163 cases of WPV type 1 have been reported worldwide in 2019, which is almost five times the total number of cases in 2018 [1]. Thus, it is clear that although the world is on the brink of eradication of all poliovirus types, the transmission of WPV continues in high-risk areas.

In addition to WPV, circulating vaccine-derived poliovirus (cVDPV) continues to be a major concern, with 242 cVDPV type 2 and eight cVDPV type 1 reported in 2019 [1]. Such risks of cVDPV and vaccine-associated paralytic poliomyelitis further highlight the importance of maintaining high immunisation coverage and the need for transition in the near future from oral polio vaccines (OPV) to inactivated polio vaccines (IPV) to complete and sustain eradication of all types of polioviruses [3]. The transition, however, is complicated by the limited number of doses of IPV, as in recent years a significant shortage in the global IPV supply has been reported. It is estimated that, in addition to the routine vaccine needs, 43 million doses will be required for catch-up immunisation in children who missed their primary vaccination due to the shortfalls [4]. AJ Vaccines has developed an aluminium hydroxide-adjuvanted IPV (IPV-Al), containing one-tenth of a standard dose of IPV. Since the antigen is the most expensive component of the vaccine, reducing its content would reduce the manufacturing cost. Thus IPV-Al can contribute to mitigating global supply and cost constraints of standard IPV.

It has previously been shown that IPV-Al is safe, immunogenic, and non-inferior to standard IPV with respect to seroconversion (non-inferiority margin of 10%) and seroprotection (non-inferiority margin of 5%) in infants vaccinated at 6, 10 and 14 weeks, according to the World Health Organization (WHO) expanded programme of immunisation (EPI) schedule [5], [6]. Furthermore, IPV-Al induced robust booster responses in these infants vaccinated at 9 months [6] and in adolescents aged 10–15 years [7]. In this paper, we present two clinical trials: a phase 3 primary vaccination trial with infants vaccinated with three doses of either IPV-Al or IPV at 2, 4 and 6 months of age, and a booster trial where the same subjects received a booster vaccination with IPV-Al at the age of 15–18 months. Given the programmatic importance of overcoming supply constraints related to IPV, the overall purpose of these trials was to demonstrate non-inferiority of IPV-Al to standard IPV and to investigate booster responses of IPV-Al in infants primed with either standard IPV or IPV-Al.

## 2 Methods

### 2.1 Trial design and subjects

The primary trial was a phase 3, non-inferiority, observer-blinded, randomised (1:1), active-controlled, multicentre clinical trial with two parallel groups of infants who received either IPV-Al (n = 400) or IPV vaccinations (n = 400) at 2, 4 and 6 months of age (a total of three primary vaccinations). Blood samples were taken prior to the first vaccination (at 2 months) and one month after the primary vaccination series (at 7 months).

The booster trial was an extension to the primary trial. It was a phase 3, open-label, multicentre clinical trial with one group (N = 666) of children who had received three doses of either IPV-Al (N = 346) or standard IPV (N = 320) in the primary trial. All subjects, whether they received IPV-Al or standard IPV as primary vaccinations, received a single booster dose of IPV-Al at the age of 15–18 months. Blood samples were collected before and one month after the booster vaccination.

Both trials were conducted at four investigational sites in Panama City, Panama. The infants were recruited through their parents/guardians during late pregnancy and/or immediately after birth. The enrolled infants in the primary trial were healthy, aged 2 months on the date of the first vaccination who had not previously been vaccinated with any polio vaccine. All subjects who completed the primary trial were offered participation in the booster trial.

Parents/guardians of the infants were informed about the trial and signed a consent allowing their child to be included and to receive the trial vaccine. They also granted access to the infant's trial-related medical records. Key exclusion criteria included known exposure to OPV or any wild or vaccine-derived poliovirus in the household within three months before inclusion (applicable to the primary trial), previous vaccination with OPV or IPV outside the primary trial (applicable to the booster trial), known or suspected allergy to the vaccine constituents, treatment with systemic corticosteroids prior to inclusion or planned during the trial period, low birth weight (<2.5 kg), known or suspected immunodeficiency or family history of congenital or hereditary immunodeficiency and severe uncontrolled chronic disease.

Trial protocols were approved by the relevant ethics committees and competent authorities prior to trial commencement. The trials were conducted according to the principles of good clinical practice [8] and the Declaration of Helsinki [9]. The trials are registered with ClinicalTrials.gov (NCT03025750: primary trial and NCT03671616: booster trial).

### 2.2 Trial vaccines

The investigational and comparator vaccines were both manufactured by Statens Serum Institut (SSI)^1^ (Copenhagen S, Denmark) and contained inactivated poliovirus types 1 (Brunhilde), 2 (MEF-1) and 3 (Saukett). The comparator vaccine was a licenced non-adjuvanted standard IPV containing 40 D-antigen units (DU) of poliovirus type 1, 8 DU of type 2 and 32 DU of type 3 per dose, and appearing as a clear solution for injection. The investigational IPV-Al was an IPV containing one tenth of the amount of each antigen present in the standard IPV, adjuvanted to aluminium hydroxide (0.5 mg aluminium), appearing as an opaque suspension for injection.

Each vaccine dose of 0.5 mL IPV-Al or IPV was administered intramuscularly, as described in detail previously [6]. Other routine childhood vaccines were administered concomitantly. In the primary trial, >99% of subjects received DTwP-HepB-Hib vaccine and rotavirus vaccine at least once, 87% received at least one dose of pneumococcal vaccine and 65% received influenza vaccine. In the extension trial 70% of subjects received influenza vaccine. The injectable concomitant vaccines were to be administered in the opposite thigh of the trial vaccines.

### 2.3 Randomisation and blinding

In the primary trial, randomisation was performed using SAS software, version 9.4 (SAS Institute, Cary, NC, USA), as described previously [6]. As the two vaccine formulations were visually different, the identity of the trial vaccine administered to a trial subject was known to prespecified site staff and the trial monitor (observer-blind design). The booster trial was an open-label trial, with all subjects receiving one dose of IPV-Al.

### 2.4 Immunogenicity endpoints

#### 2.4.1 Primary trial

The primary endpoint was the proportion of infants with type-specific seroconversion one month after the primary vaccination series, defined as 1) a post-vaccination antibody titre ≥4-fold higher than the estimated maternal antibody titre, based on the pre-vaccination titre declining by a half-life of 28 days, and 2) a post-vaccination titre ≥8. The established correlate of protection against polio is a neutralising antibody titre of ≥8 for the poliovirus types 1, 2 and 3 [10], [11], [12].

Secondary immunogenicity-related endpoints included the proportion of infants with seroprotection (antibody titre ≥8) against poliovirus types 1, 2 and 3, respectively, one month after the primary vaccination series. Geometric mean titres (GMTs) were also analysed and the proportion of infants with post-vaccination titres ≥4-fold higher than the estimated maternal antibody titre.

Exploratory endpoints included the same immunogenicity endpoints as for the primary analysis, but analysed in subgroups of infants with (titre ≥8) and without (titre <8) seroprotection at baseline (at 2 months of age).

#### 2.4.2 Booster trial

The primary endpoint was the type-specific (poliovirus types 1, 2 and 3) booster effect (GMT post-booster (day 28)/ GMT pre-booster (day 0)) calculated from individual serum titre values.

The secondary endpoints included the proportion of children with seroprotection (antibody titre ≥8) and GMTs against poliovirus types 1, 2 and 3 before and one month after the booster vaccination. Antibody persistence, defined as relative individual serum titre changes from the end of the primary vaccination series until just before the booster vaccination was also analysed.

A validated Vero cell assay was used for the antibody titre determinations in both trials and has been described previously [7], [13]. The lower limit in the measurable range of titres was 1.4 and there was no upper limit.

### 2.5 Safety endpoints

Safety endpoints in both trials were solicited injection site adverse events (AEs) and systemic AEs days 0–3 and, only if detection of AEs, days 4–6 following the vaccinations (collected through parent electronic diaries), as well as all other reported AEs while in the trial. In the primary trial, there was also a safety follow-up by phone at the age of 12 months. The defined solicited injection site AEs and systemic AEs were described previously [6].

### 2.6 Statistical analyses

SAS software, version 9.4, was used for the analyses in both trials.

#### 2.6.1 Primary trial

A non-inferiority margin of 10% for seroconversion and 5% for seroprotection were selected for the primary and secondary immunogenicity endpoints, respectively. The choice of margins has been described previously [6].

The sample size calculation was based on seroconversion rates (ranging from 94.6% to 100%) in a phase 2 dose-investigation clinical trial [5]. If the true seroconversion for IPV-Al was lower than for IPV, e.g. by 5%-point for all three poliovirus types, then 350 subjects per treatment group were considered sufficient to achieve a power of >80%. Based on these data and a non-inferiority margin of 10% for the primary immunogenicity endpoint analysis and allowance for possible exclusion of up to 15% from the primary immunogenicity analysis, it was concluded that it would be sufficient to include 400 subjects per arm.

Detailed descriptions of the primary and secondary immunogenicity analysis have been provided previously [6]. The estimated maternal antibody titre was calculated as:

Titre(t) = titre (baseline)×exp (-(ln(2)/t1/2)×t)

where t was the time in days since baseline and t½ was the expected half-life of maternal antibodies of 28 days

The primary immunogenicity analysis was based on the per-protocol (PP) analysis set (defined as all subjects in the full analysis set (FAS) who had no major protocol deviations). Sensitivity analyses of the primary endpoint were performed using the FAS (defined as all randomised and vaccinated infants with at least one post-baseline measurement). All secondary immunogenicity analyses were based on both PP and FAS. The proportion of infants with seroprotection (titre ≥8) against poliovirus types 1, 2 and 3, one month after the primary vaccination series was analysed using the same method as used for the primary immunogenicity endpoint, but with a 5%-points non-inferiority margin. Geometric mean and median titres for poliovirus types 1, 2 and 3 as well as safety endpoints were summarised using descriptive statistics. The exploratory endpoints were analysed similarly to the primary and secondary endpoints except that non-inferiority assessments were not conducted. The analysis of safety endpoints was based on the safety analysis set (SAF), defined as all subjects who received at least one trial vaccination.

#### 2.6.2 Booster trial

Since the booster trial was an extension to the primary trial, no independent sample size determination was performed. Summaries for the primary and secondary endpoints were presented both for the FAS-booster (defined as all enrolled subjects with the primary endpoint, i.e. the booster effect, for at least one of the three poliovirus types) and PP-booster (defined as all FAS subjects who did not have major deviations from the protocol) analysis set. Analyses of safety endpoints were based on SAF-booster set, defined as all subjects who received the booster vaccination. All analysis was descriptive in form.

## 3 Results

### 3.1 Population characteristics

#### 3.1.1 Primary trial

**Fig. 1:**
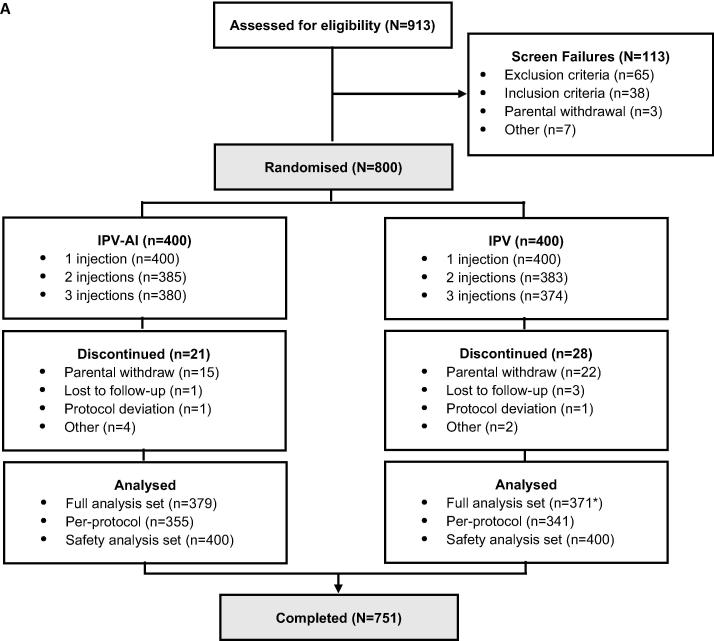

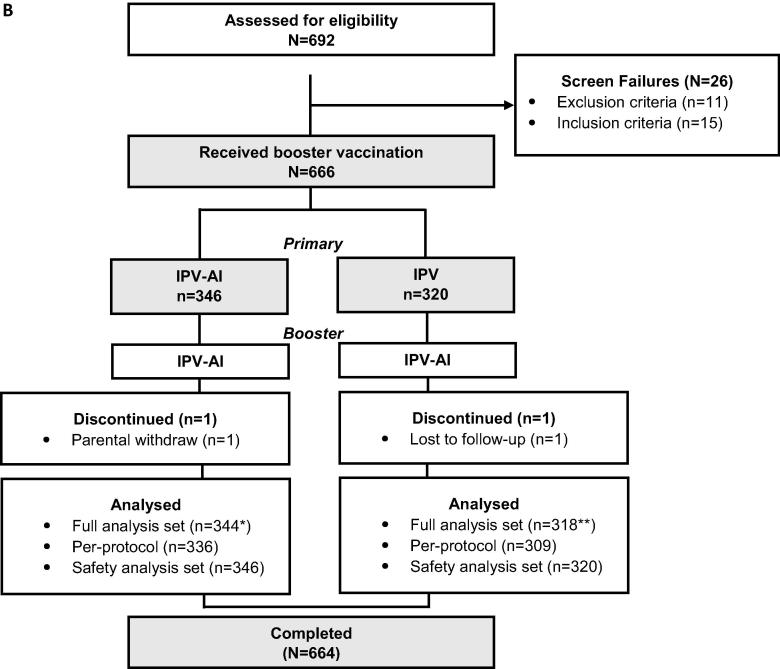
Disposition of subjects in the primary (A) and booster (B) trial. Fig. 1A. The full analysis set (FAS) defined as all randomised and vaccinated infants with at least one post-baseline measurement. The per-protocol (PP) analysis set was defined as all infants in the FAS who had no major deviations from the protocol. The safety analysis set (SAF) was defined as all randomised infants who received at least one treatment dose. N = number of infants in the group. *One subject completed but did not have a sample post-primary vaccination series and was excluded from FAS (hence FAS = 371). Fig. 1B. The full analysis set (FAS-booster) was defined as all enrolled subjects with the primary endpoint, i.e. the booster effect, for at least one of the three poliovirus types. The per-protocol analysis set (PP) was defined as all FAS subjects who did not have major deviations from the protocol. The safety analysis set (SAF) was defined as all enrolled subjects who received at least one trial vaccination. *Two subjects primed with IPV-Al did not have post-booster samples due to discontinuation and difficult venous access. ** Two subjects primed with IPV did not have post-booster samples due to discontinuation and difficult venous access.

The trial was conducted between 19 January 2017 (first trial visit) and 14 November 2017 (last trial visit). 913 potential subjects were screened and assessed for eligibility, of which 113 were screen failures (Fig. 1A). The enrolled infants (N = 800) were randomised to receive either IPV-Al (n = 400) or the comparator IPV (n = 400). 751 infants (94%) completed the trial visit one month after the primary vaccination series, of which one subject from the IPV group did not have vaccination sample after the primary vaccination series and thus was not included in the FAS.

54 subjects were excluded from the PP due to major protocol deviations. For 41 of these subjects, the major deviation was related to systemic receipt of prednisolone while in the trial. As a result, 696 subjects were included in the PP. Both vaccination groups were comparable in terms of demography and baseline characteristics (Table 1).

**Table 1:** Baseline characteristics of the primary and booster trial population.

Characteristics	Variable	Primary trial		Booster trial	
		IPV-Al	IPV	IPV-Al-primed	IPV- primed
	N	400 (100.0)	400 (100.0)	346 (100.0)	320 (100.0)
Sex, n (%)	Female	203 (50.8)	181 (45.3)	176 (50.9)	147 (45.9)
	Male	197 (49.3)	219 (54.8)	170 (49.1)	173 (54.1)
Age (days)	Mean (SD)	58.13 (4.82)	58.11 (4.82)	507.52 (34.37)	505.76 (34.32)
	Min ; Max	54 ; 75	54 ; 75	456 ; 561	456 ; 561
Birth weight (kg)	Mean (SD)	3.29 (0.41)	3.31 (0.41)	3.28 (0.41)	3.32 (0.42)
	Min ; Max	2.5 ; 4.71	2.5 ; 4.8	2.5 ; 4.71	2.5 ; 4.8
Race, n (%)	Latin American	282 (70.5)	271 (67.8)	244 (70.5)	225 (70.3)
	American Indian or Alaska Native	97 (24.3)	106 (26.5)	83 (24.0)	76 (23.8)
	Black or African American	14 (3.5)	15 (3.8)	12 (3.5)	12 (3.8)
	White	7 (1.8)	8 (2.0)	7 (2.0)	7 (2.2)

Data are n (%) or mean (SD) for subjects in the safety analysis set (SAF).

N = number of subjects in trial group; n(%) = number (percentage) of subject with data; SD = standard deviation;

#### 3.1.2 Booster trial

The trial was conducted between 15 May 2018 (first trial visit) and 03 October 2018 (last trial visit). Of the 800 subjects who participated in the primary trial, 692 subjects agreed to participate in the extension trial. All were screened and assessed for eligibility, resulting in 26 screen failures (Fig. 1B). 666 subjects were enrolled in the trial and received a single dose of IPV-Al as a booster vaccination. 664 subjects were included in the FAS and 645 subjects in the PP.

Basic demographic information was collected during the primary trial and was transferred to the booster trial after the database had been locked. There were no significant differences in demography and baseline characteristics between subjects primed with IPV-Al and IPV (Table 1).

### 3.2 Immunogenicity results

#### 3.2.1 Primary trial

Seroconversion rates (primary endpoint) after vaccination with three doses of IPV-Al were 96.1% (type 1), 100% (type 2) and 99.2% (type 3) while all subjects (100%) obtained seroconversion in the IPV group. Non-inferiority of IPV-Al to standard IPV with respect to the primary endpoint of seroconversion was demonstrated for each of the three poliovirus types, as indicated by the lower limit of the two-sided 95% confidence intervals (CIs) of the rate differences being above the pre-defined limit of −10%-points (Table 2).

**Table 2:** Non-inferiority analysis of seroconversion (primary endpoint) and secondary endpoint seroprotection (titre ≥8) rates one month after the primary vaccination series.

Poliovirus types	IPV-Al				IPV				% Difference	
	N	n	%	95% CI	N	n	%	95% CI	Diff	95% CI
**Seroconversion**										
Type 1	355	341	96.1	93.5 ; 97.8	341	341	100.0	98.9 ; 100.0	−3.94	−6.51 ; −2.01
Type 2	355	355	100.0	98.4 ; 100.0	341	341	100.0	98.4 ; 100.0	0.00	−1.30 ; 1.37
Type 3	354	351	99.2	97.5 ; 99.8	341	341	100.0	98.9 ; 100.0	−0.85	−2.46 ; 0.40

**Seroprotection**										
Type 1	355	343	96.6	94.2 ; 98.2	341	341	100.0	98.9 ; 100.0	−3.38	−5.81 ; −1.56
Type 2	355	355	100.0	98.4 ; 100.0	341	341	100.0	98.4 ; 100.0	0.00	−1.30 ; 1.37
Type 3	354	351	99.2	97.5 ; 99.8	341	341	100.0	98.9 ; 100.0	−0.85	−2.46 ; 0.40

Data are presented for the per-protocol (PP) analysis set. The primary endpoint was defined as 1) a type-specific post-vaccination titre ≥4-fold higher than the estimated maternal antibody titre, based on the pre-vaccination titre declining by a half-life of 28 days, and 2) a type-specific post-vaccination titre ≥8.

CI = confidence interval; Diff = difference; N = number of subjects in group; n = number of subjects with seroconversion; %=percentage of subjects with seroconversion

In the sensitivity analysis of the primary seroconversion endpoint in the FAS, no major difference between the PP and the FAS population results was observed, and non-inferiority of IPV-Al to standard IPV was also demonstrated in the FAS.

The proportion of infants with post-vaccination titres ≥4-fold above the estimated maternal antibody titres (secondary endpoint) were 99.4% (IPV-Al) and 100% (IPV) for type 1, and 100% in both trial groups for type 2 and type 3. In addition, between 96.6 and 100% of infants in the IPV-Al group and 100% in the IPV group were seroprotected (antibody titres ≥8) after the primary vaccination series (Table 2). Non-inferiority for seroprotection rates was confirmed for IPV-Al for poliovirus type 2 and type 3, as the seroprotection rates for the two types were less than 5%-point lower than for IPV. However, non-inferiority for seroprotection was not confirmed for IPV-Al for poliovirus type 1 as the lower confidence limit was −5.81.

At baseline at age 2 months GMTs were similar between the two groups (Table 3). Post-primary vaccination GMTs were high for all poliovirus types in both IPV-Al and IPV groups (Table 3), with higher GMTs across all poliovirus types observed after priming with IPV.

**Table 3:** Summary of seroprotection rates and serum neutralising antibody titres for poliovirus types 1, 2 and 3 reported in the primary and booster trial.

Poliovirus type and endpoint	Baseline (age 2 months)		Post-primary vaccination (age 7 months)		Pre-booster vaccination (age 15–18 months)		Post-booster vaccination (age 16–19 months)	
	IPV-Al n=355	IPV n=341	IPV-Al n=355	IPV n=341	IPV-Al-primed n=336	IPV-primed n=309	IPV-Al-primed n=336	IPV-primed n=309
**Type 1**								
Seroprotection (titre ≥8), %	61.4	58.9	96.6	100.0	83.6	99.4	97.0	100.0
GMT (95% CI)	11.9 (10.2; 13.9)	10.4 (9.0; 12.0)	809.3 (656; 999)	4775 (4321; 5278)	96.9 (76; 123)	557.6 (486; 639)	2456 (1935; 3117)	5149 (4655; 5695)
Median	11.3	8.0	1448	5793	181	512	4096	5793

**Type 2**								
Seroprotection (titre ≥8), %	86.8	88	100.0	100.0	100.0	100.0	100.0	100.0
GMT (95% CI)	34.0 (29.3; 39.5)	35.7 (30.9; 41.3)	3256 (2909; 3645)	6934 (6244; 7701)	469.0 (416; 529)	1190 (1038; 1365)	8980 (7900; 10206)	7728 (6985; 8550)
Median	32.0	32.0	2896	5793	512	1024	8192	8192

**Type 3**								
Seroprotection (titre ≥8), %	50.1	47.8	99.2	100.0	78.9	98.4	100.0	100.0
GMT (95% CI)	8.2 (7.1; 9.4)	8.1 (7.0; 9.4)	786.9 (648; 956)	4785 (4249; 5389)	63.0 (49; 80)	425.5 (357; 507)	3172 (2610; 3853)	5301 (4740; 5930)
Median	8.0	5.7	1024	5793	91	512	4096	5793

Data are presented for the per-protocol (PP) analysis sets. Not all infants from the primary trial proceeded to the extension (booster) trial. All subjects in the extension trial were boosted with IPV-Al. CI = confidence interval, GMT = geometric mean titre, n = number of infants in trial group.

The explorative comparisons between the two subgroups – those with seroprotection at baseline and those without seroprotection at baseline – demonstrated high seroconversion (primary endpoint) (>93%) and seroprotection (>93%) after the primary vaccination series for all poliovirus types irrespective of their seroprotection status at baseline and vaccine received.

#### 3.2.2 Booster trial

The booster effects (GMT post-booster / GMT pre-booster) for the IPV-Al-primed group compared with the IPV-primed group were: 25.3 (95% CI 21.7; 29.5) vs 9.2 (7.9; 10.8) for type 1, 19.1 (16.8; 21.8) vs 6.5 (5.7; 7.4) for type 2 and 50.4 (42; 60.5) vs 12.5 (10.3; 15.1) for type 3. When correcting for the pre-booster levels, booster effects for the group primed with IPV-Al compared to the group primed with IPV were 18.7 vs 12.9 (type 1), 14.6 vs 8.7 (type 2) and 29.3 vs 22.4 (type 3). The booster effects in the FAS population were almost identical to those in the PP population.

The pre-booster GMTs were lower in the group primed with IPV-Al than with IPV for all poliovirus types (Table 3). After the booster vaccination, GMTs for the group primed with IPV-Al were lower than for the group primed with IPV for type 1 and type 3, whereas for type 2 the post-booster GMTs were comparable between the two groups (Table 3).

Before the booster vaccination, the seroprotection rates (antibody titre ≥8) in the PP population were lower in the group primed with IPV-Al than with IPV for poliovirus type 1 and type 3, and comparable to IPV for type 2 (Table 3). The post-booster seroprotection rates in the PP population were 97% (IPV-Al-primed group) and 100% (IPV-primed group) for type 1, and all subjects (100%) were seroprotected against type 2 and type 3. Ten subjects (3%) primed with IPV-Al were not seroprotected against poliovirus type 1 after the booster vaccination. Five of these were not seroprotected after the primary vaccination series.

**Fig. 2:**
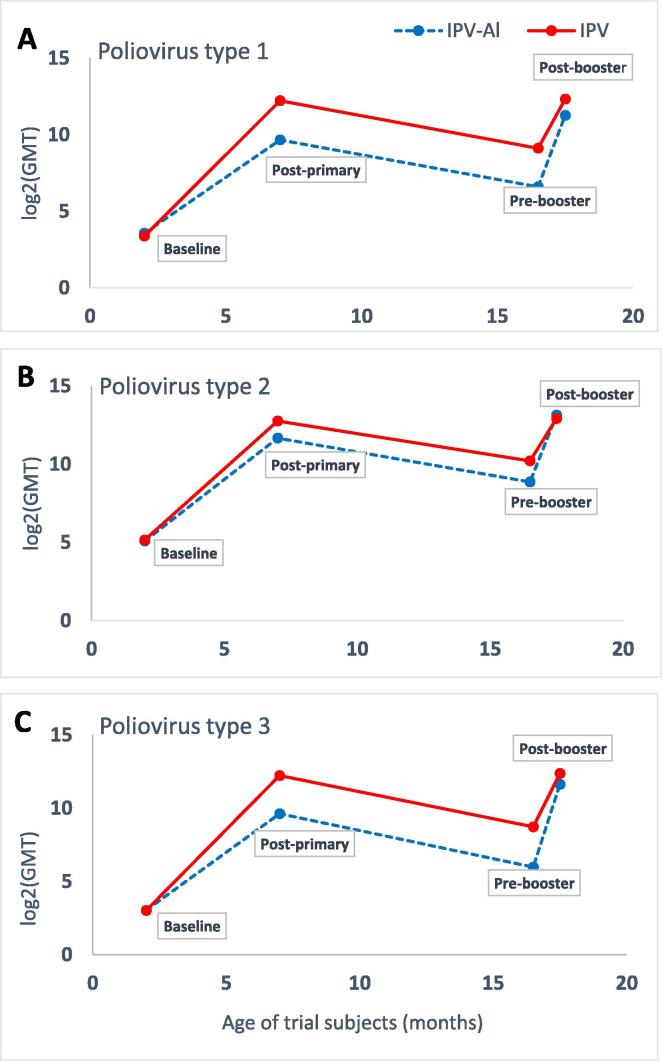
Geometric mean titres (GMTs) in infants primed with IPV-Al and IPV at baseline (2 months), post-primary vaccination (7 months), pre-booster (mean 16.5 months, range 15–18 months) and post-booster (mean 17.5 months, range 16–19 months) for poliovirus type 1 (A), type 2 (B) and type 3 (C). GMTs are shown as log2 values.

Antibody persistence analysis for both priming groups showed a decline in GMTs from post-primary vaccination until the pre-booster vaccination across all poliovirus types (Table 3 and Fig. 2). The post-primary and pre-booster vaccination GMTs for all poliovirus types were lower in the group primed with IPV-Al than with IPV. The seroprotection rates for both groups were lower at pre-booster than at post-primary vaccination for type 1 and type 3, while the rates were 100% for type 2 in both groups.

**Fig. 3:**
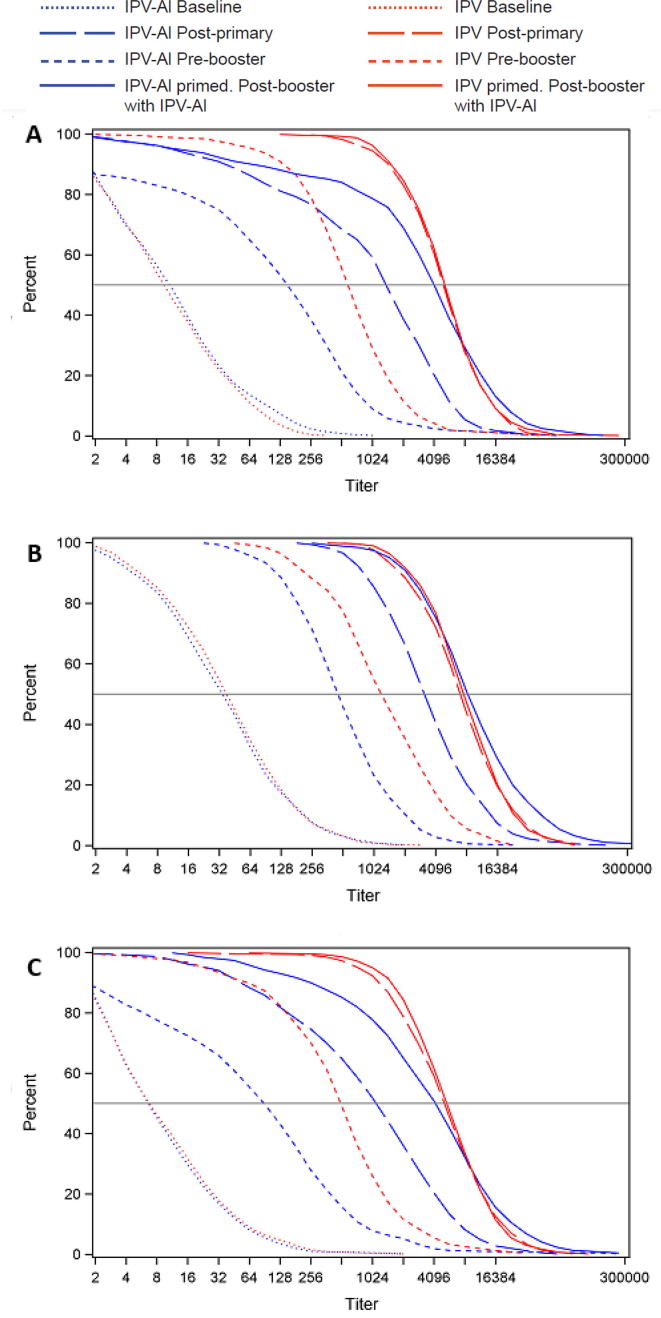
Reverse cumulative titre distribution at baseline (2 months), post-primary vaccination (7 months), pre-booster (15–18 months) and post-booster (16–19 months) for poliovirus type 1 (A), type 2 (B) and type 3 (C). Subjects received either IPV-Al or IPV as primary vaccination; all subjects received IPV-Al as booster vaccination.

Reverse cumulative distribution curves illustrate changes in antibody titre distributions for the four timepoints, i.e. pre-primary, post-primary, pre-booster and post-booster vaccination (Fig. 3A–C). The post-primary vaccination curves show that both vaccines were immunogenic with similar curve shapes for IPV-Al and IPV across poliovirus types. Consistent with the higher antigen content in IPV, the post-primary vaccination curves for IPV are right-shifted compared to the curves for IPV-Al. The pre-booster vaccination curves illustrate the decline in antibody levels from post-primary vaccination to pre-booster vaccination, with the rates of decline similar in both groups for the respective poliovirus types. The post-booster titre distribution was similar in the IPV-Al and IPV-primed groups and increases in antibody titres were observed for all poliovirus types in both groups.

### 3.3 Safety results

#### 3.3.1 Primary trial

The proportion of infants with at least one AE reported by their parents/guardians during the trial was similar across groups: 96.3% in the IPV-Al group vs 95.3% in the IPV group. No AEs with fatal outcome were reported during the trial. In total, 46 serious AEs were recorded: 19 events in 16 infants from the IPV-Al group (4%) and 27 events in 21 infants from the IPV group (5.3%). The most common serious AEs were pneumonia and bronchiolitis. All serious AEs were assessed as not related to the trial vaccine and had resolved by the end of the trial.

The proportion of infants with at least one systemic AE was also similar between the IPV-Al (96%) and IPV (95.0%) vaccine groups (Table 4). The three most common systemic AEs were crying, pyrexia and nasopharyngitis. The majority of the systemic events were of mild intensity (78.4%), while the rest were moderate (19.2%) or severe (2.4%). Overall, the proportion of infants with at least one injection site reaction was similar between the IPV-Al (16.5%) and IPV (12.5%) vaccine groups (Table 4). Injection site reactions reported were either injection site redness or swelling, and they were either of mild or moderate intensity. 85% of redness and swelling were <25 mm in diameter, 10% were ≥25 mm, and 5% were not measured.

**Table 4:** Summary of solicited adverse injection site reactions and systemic adverse events, and other adverse events with a frequency of at least 5% of subjects in any group, reported in the primary trial and booster trial.

Adverse event type	Primary trial		Booster trial	
	IPV-Al N=400 n (%) e	IPV N=400 n (%) e	IPV-Al-primed N=346 n (%) e	IPV-primed N=320 n (%) e
**Mild adverse injection site reactions**	63 (15.8) 90	47 (11.8) 75	9 (2.6) 13	5 (1.6) 6
Injection site erythema	51 (12.8) 52	35 (8.8) 41	9 (2.6) 9	5 (1.6) 5
Injection site swelling	33 (8.3) 38	29 (7.3) 34	4 (1.2) 4	1 (0.3) 1
**Moderate adverse injection site reactions**	6 (1.5) 6	8 (2.0) 8	–	–
Injection site swelling	3 (0.8) 3	5 (1.3) 5		
Injection site erythema	3 (0.8) 3	3 (0.8) 3		
**AEs with frequency ≥5%**	384 (96) 2058	380 (95.0) 2158	124 (35.8) 198	135 (42.2) 204
Crying	191 (47.8) 295	206 (51.5) 307	15 (4.3) 16	18 (5.6) 19
Pyrexia	183 (45.8) 259	199 (49.8) 282	17 (4.9) 17	16 (5.0) 16
Nasopharyngitis	170 (42.5) 254	165 (41.3) 248	46 (13.3) 47	54 (16.9) 55
Tonsilitis	123 (30.8) 206	117 (29.3) 190		
Irritability	120 (30.0) 192	123 (30.8) 206	24 (6.9) 24	17 (5.3) 17
Somnolence	106 (26.5) 148	94 (23.5) 129		
Decreased appetite	87 (21.8) 113	79 (19.8) 110		
Vomiting	66 (16.5) 77	67 (16.8) 83		
Body temperature inc.	36 (9.0) 41	41 (10.3) 45		
Dermatitis diaper	32 (8.0) 38	40 (10.0) 43		
Rhinitis	30 (7.5) 33	42 (10.5) 45		
Microcytic anaemia	29 (7.3) 29	37 (9.3) 37		
Acarodermatitis	23 (5.8) 25	29 (7.3) 30		
Bronchiolitis	23 (5.8) 27	28 (7.0) 33		
Diarrhoea	18 (4.5) 20	30 (7.5) 31		
Conjunctivitis	25 (6.3) 25	24 (6.0) 25		
Gastroenteritis	20 (5.0) 20	28 (7.0) 31	8 (2.3) 8	16 (5.0) 16

Data are for subjects in the safety analysis sets (SAF). AEs are presented by MedDRA (version 19.1) preferred term.

AE = adverse event, e = number of events, MedDRA = Medical Dictionary for Regulatory Activities, N = number of infants in group, n (%) = number (percentage) of infants with AE, URTI, upper respiratory tract infection.

* Solicited AEs in both trails were as follows: injection site redness/swelling, axillary temperature, abnormal crying, irritability, drowsiness, loss of appetite and vomiting.

#### 3.3.2 Booster trial

A total of 421 AEs were reported in 269 (40.4%) subjects, of which 98.1% of AEs were non-serious, and 1.9% (eight events) were serious. The eight serious AEs were reported in seven subjects: three subjects primed with IPV-Al and four primed with IPV. The serious AEs included pneumonia (two events), gastroenteritis (two events), vomiting, diarrhoea, ileus paralytic and burn second degree. Two serious AEs (ileus paralytic and diarrhoea) were assessed as possibly related to the vaccine. All subjects with serious AEs recovered without sequelae.

The proportion of subjects with at least one systemic AE was higher in the group primed with IPV (42.2%) than with IPV-Al (35.8%). The most common systemic AEs reported were nasopharyngitis, irritability, crying, pyrexia and gastroenteritis (Table 4). Most of the systemic AEs were of mild intensity (89.3%), whereas the rest were moderate (9.5%) or severe (1.2%).

The proportion of subjects with at least one injection site reaction was higher in the group primed with IPV-Al (2.6%) than with IPV (1.6%) (Table 4). All injection site reactions were redness or swelling, and all were of mild intensity (<25 mm).

## 4 Discussion

This phase 3 primary trial showed that the dose-sparing IPV-Al was non-inferior to full-dose IPV with regards to seroconversion rates (non-inferiority margin of 10%) for poliovirus type 1, 2 and 3, in healthy Panamanian infants one month after vaccination at 2, 4 and 6 months of age. Furthermore, IPV-Al was non-inferior to standard IPV with respect to seroprotection rates for poliovirus type 2 and 3, with a pre-defined non-inferiority margin of 5%. For poliovirus type 1 the lower 95% confidence limit was just below the limit (-5.81%). Taking into account the low risk of infection with poliovirus [1], the seroprotection rate of 96.6% for IPV-Al for type 1 is considered high. These results confirm the findings from two recent clinical trials carried out in the Dominican Republic [5] and the Philippines [6], where IPV-Al post-primary vaccination seroconversion and seroprotection rates were non-inferior to those of standard IPV in healthy infants one month after vaccination according to the EPI schedule (vaccination at 6, 10 and 14 weeks of age). The seroprotection rates obtained in this trial for IPV-Al (96.6%–100%) were similar to those reported (96.9%–100%) for penta- and hexavelant primary vaccines containing adjuvanted full-dose IPV administered at 2,4 and 6 months of age [14], [15], [16], [17].

The booster responses of IPV-Al were previously investigated in two other trials: a phase 3 trial in the Philippines, in infants (primed with IPV-Al according to the EPI schedule) and boosted at the age of 9 months [6], and a phase 2 trial in Danish adolescents boosted at 10–15 years of age with a vaccination history of IPV at 3, 5, 12 months and 5 years of age [7]. Overall, the post-booster GMTs for IPV-Al were high and above the seroprotection threshold in all three trials, demonstrating the ability of IPV-Al to boost responses in both IPV-Al and IPV-primed subjects. Some differences were observed between the trials: the booster effects and post-booster GMTs obtained after vaccination with IPV-Al in the present trial were lower than reported in the infant trial in the Philippines, whereas the booster effects for IPV-Al in the adolescent trial in Denmark were within the range of the present trial. The booster effects and post-booster GMTs for IPV-Al in the present trial were comparable to booster responses of adjuvanted full-dose IPV observed in two recent trials in China and UK, in children boosted at 18–24 months [18] and 3–4 years of age [19]. In addition, the seroprotection rates obtained for a booster dose of IPV-Al in the present and the two previous trials (97–100%) are in line with seroprotection rates reported for licensed vaccines containing adjuvanted full-dose IPV given as booster vaccinations in the second year of life (≥98.5%) [20], [21].

Similar to what was observed in the previous two trials, the post-primary vaccination GMT levels were lower for IPV-Al than for IPV. This was expected due to the lower antigen content in IPV-Al. However, these lower levels are not expected to be clinically relevant since both the post-primary and pre- and post-booster vaccination GMT levels for IPV-Al were well above the seroprotection limit of 8 (Table 3).

In the present trial, ten infants (3%) primed with IPV-Al did not have protective titres (≥8) for poliovirus type 1 after the booster vaccination. Five of these infants seroconverted and obtained seroprotection (titres between 11 and 64) after the primary vaccination series. As the ability of a booster vaccine to mount an adequate response depends on the immune memory (generation of B memory cells) established during the primary vaccination [22], it appears that these five infants may have failed to develop immune memory, even though there was an observable immunological response following primary vaccination. For the remaining five infants titres remained below eight at all post-vaccination time points.

The rate of decline in GMT levels from the primary to the booster vaccination observed in the present trial was lower than that in the Philippine trial irrespective of the vaccines and poliovirus types. The length of time from the last primary to the booster vaccination was longer in this trial than in Philippine trial, thus suggesting exponential decline over time. These results are in line with the antibody decline observed by others [23], [24], [25], [26]. Even though the post-primary vaccination GMTs for IPV were higher than those for IPV-Al, the rate of decline of type-specific antibody levels was similar for both vaccination groups; this was observed both in the present trial and in the trial carried out in the Philippines.

Consistent with declining antibody titres, the seroprotection rates for poliovirus types 1 and 3 in the IPV-Al primed group fell to 83.6% and 78.9%, respectively, at the time of the booster vaccination. Following the booster vaccination, 97–100% of subjects were seroprotected (titre ≥8) against all poliovirus types. These results, combined with the clear booster effects and post-booster GMTs higher than post-primary GMTs, indicate that efficient immune memory was established in almost all subjects after their primary vaccination series with IPV-Al. Consequently, should a titre level fall below the protective threshold over time, subsequent antigenic re-stimulation (from vaccine or poliovirus) is expected to rapidly induce a return of protective antibody levels in the vast majority of the population vaccinated with IPV-Al [22].

IPV-Al was well tolerated in both the primary and the booster trial. No fatal cases were reported during the trials. Only two serious events in the booster trial (ileus paralytic and diarrhoea) were assessed as possibly related to the vaccine. No differences in the frequencies of AEs between the IPV-Al and IPV groups were observed. These results, together with safety data from the previous three trials with IPV-Al [5], [6], [7] and other trials with aluminium hydroxide-adjuvanted IPV in different combinations [27], [28], [29], [30] do not suggest any safety concerns following the addition of aluminium hydroxide to IPV.

The results from the presented two trials support the findings from the previous four trials with IPV-Al, which demonstrated that IPV-Al is safe and immunogenic when administered as a primary and booster vaccine. The six clinical trials with IPV-Al were conducted in Panama, the Philippines, the Dominican Republic and Denmark. Despite the geographical differences, the baseline (pre-primary) GMTs were consistent for each poliovirus type across the three primary vaccination trials, with the pre-vaccination type 2 titres being higher than type 1 and 3 titres. The post-vaccination results were also comparable across trials. The consistency of results across different parts of the world supports the general understanding that the immunogenicity profile of IPV is unaffected by socio-demographic or geographic factors [31] and allows for extrapolation of the results to other regions.

The applicability of IPV-Al as a primary vaccine was assessed in two different primary schedules: the 2, 4 and 6 month-schedule (presented here) and the 6, 10 and 14 week-schedule (EPI-schedule trials in the Dominican Republic and the Philippines). The EPI schedule can be challenging with respect to age at first vaccination and the short interval between vaccinations. The first vaccination is given early (6 weeks) with high levels of maternal antibodies, which might interfere with serological responses in the infants [32], [33]. For poliovirus type 2, the serological responses were higher in the 2, 4 and 6 month-schedule than in the EPI schedule, whereas for poliovirus types 1 and 3 the immunological results were similar in the two schedules. As the seroprotection and seroconversion rates were high in both schedules, it is expected that IPV-Al would also be immunogenic in less condensed/challenging schedules.

In addition to the knowledge already generated for IPV-Al, it would be interesting to investigate the applicability of IPV-Al in other schedules. Previous research suggests that the provision of standard IPV to OPV-vaccinated children can boost intestinal immunity and reduce viral excretion, but that IPV given alone does not seem to induce mucosal responses [34], [35]. Similar to other inactivated polio vaccines it is highly unlikely that IPV-Al will induce intestinal immunity in the absence of OPV. It would therefore be relevant, in a future study, to delineate the potential of IPV-Al for boosting intestinal immunity in OPV-vaccinated subjects. Given the GPEI recommendations for sequential cessation of OPV, there will be a time when IPV-Al will be used in a mixed vaccination schedule with bivalent OPV. Furthermore, long-term monitoring of IPV-Al vaccinated populations would be informative with regards to long-term protection.

## 5 Conclusions

Development of new IPV vaccines that can help secure a stable supply of affordable IPV to meet the increasing global demand is of critical importance for the long-term success of the global polio eradication program. Overall, six clinical trials carried out across different geographical settings demonstrated that IPV-Al is safe and immunogenic when used as a primary and booster vaccine. IPV-Al, with its reduced antigen content, has the potential for mitigating the cost and supply constraints related to the use of standard IPV. Thereby it could be an integral tool to achieve and sustain the goal of a polio-free world.

## Declaration of Competing Interest

The authors declare that they have no known competing financial interests or personal relationships that could have appeared to influence the work reported in this paper.

## Footnotes

1 The development of IPV-Al was initiated by Statens Serum Institut (SSI). Upon its establishment in January 2017, the development of IPV-Al was transferred to AJ Vaccines A/S.
